# The Mechanism of Variegation in *immutans* Provides Insight into Chloroplast Biogenesis

**DOI:** 10.3389/fpls.2012.00260

**Published:** 2012-11-27

**Authors:** Andrew Foudree, Aarthi Putarjunan, Sekhar Kambakam, Trevor Nolan, Jenna Fussell, Gennady Pogorelko, Steve Rodermel

**Affiliations:** ^1^Department of Genetics, Development, and Cell Biology, Iowa State UniversityAmes, IA, USA

**Keywords:** IMMUTANS, PTOX, variegation, chloroplast, photosynthesis, carotenoids, chloroplast biogenesis, retrograde signaling

## Abstract

The *immutans* (*im*) variegation mutant of *Arabidopsis* has green and white-sectored leaves due to the absence of fully functional plastid terminal oxidase (PTOX), a plastoquinol oxidase in thylakoid membranes. PTOX appears to be at the nexus of a growing number of biochemical pathways in the plastid, including carotenoid biosynthesis, PSI cyclic electron flow, and chlororespiration. During the early steps of chloroplast biogenesis, PTOX serves as an alternate electron sink and is a prime determinant of the redox poise of the developing photosynthetic apparatus. Whereas a lack of PTOX causes the formation of photooxidized plastids in the white sectors of *im*, compensating mechanisms allow the green sectors to escape the effects of the mutation. This manuscript provides an update on PTOX, the mechanism of *im* variegation, and findings about *im* compensatory mechanisms.

## Introduction

Variegation mutants provide an excellent system to explore the mechanisms of chloroplast biogenesis and the nature of communication between the nucleus-cytoplasm, chloroplast, and mitochondrion (Rodermel, 2001; Yu et al., 2007). The leaves of these mutants contain green and chlorotic (white, yellow) sectors that arise as a consequence of mutations in nuclear or organelle genes (Tilney-Bassett, 1975). Whereas cells in the green sectors have morphologically normal chloroplasts, cells in the chlorotic sectors contain pigment-deficient plastids with abnormal membrane structures (Aluru et al., 2001). One common mechanism of variegation involves the induction of defective chloroplasts (or mitochondria) by mutations in nuclear genes that code for organelle proteins. In some cases the green and white cells have different genotypes. For example, transposable element activity can inactivate a gene required for normal chloroplast function in some cells (chlorotic cells) while excision can reconstitute gene function (green cells). In other cases of variegation, the two types of cells have the same (mutant) genotype, indicating that the gene product is required in some, but not all cells of the mutant. This raises the question of how the green cells escape this requirement. Is there a compensating activity? Our operating hypothesis is that answers to this question will provide insight into mechanisms of variegation, as well as into pathways and regulatory networks of chloroplast biogenesis.

The past decade has witnessed a growing interest in the use of variegation mutants to address fundamental questions in photosynthesis and chloroplast biogenesis (Yu et al., 2007; Liu et al., 2010). Yet, mechanisms of variegation are still poorly understood. Here we focus on the mechanism of variegation in *immutans* (*im*), which defines the gene for plastid terminal oxidase (PTOX), a versatile quinol oxidase in plastid membranes. Early studies led to the notion that this mechanism involves the attainment of a threshold by developing plastids (the threshold hypothesis). In this manuscript, we review the underlying assumptions and experiments in support of this hypothesis and discuss approaches and studies that have been used to gain insight into factors that compensate for a lack of PTOX.

## Immutans

The *im* variegation mutant is one of the oldest *Arabidopsis* mutants, reported independently by Rédei and Röbbelen in the 1960’s (Rédei, 1963; Röbbelen, 1968). Cells in the green sectors of *im* have morphologically normal chloroplasts, whereas cells in the white sectors are heteroplastidic and contain abnormal plastids that lack pigments and organized lamellae, as well as rare, normal-appearing chloroplasts (Wetzel et al., 1994). The extent of white sector formation in *im* is promoted by high light and low temperature (Rédei, 1963, 1967; Röbbelen, 1968; Wetzel et al., 1994; Rosso et al., 2009). HPLC analyses showed that the white sectors accumulate the colorless C_40_ carotenoid intermediate, phytoene, indicating that *im* is impaired in the activity of phytoene desaturase (PDS), the enzyme which converts phytoene to zeta-carotene (Wetzel et al., 1994). All the steps of carotenogenesis take place in the plastid and are mediated by nuclear-encoded enzymes that are imported into the organelle post-translationally, and PDS mediates an early step of the pathway (DellaPenna and Pogson, 2006). An inhibition of PDS activity would thus result in lack of accumulation of downstream, colored (photoprotective) carotenoids, and under high light conditions, would be anticipated to give rise to white, photooxidized plastids. A defect of this sort is consistent with the light-sensitivity of *im*; as in other carotenoid mutants, the higher the light intensity, the greater the extent of photooxidation (white sector formation) (Oelmüller, 1989; Mayfield and Taylor, 2005). The presence of normal-appearing chloroplasts in *im*, on the other hand, suggests that some plastids are able to bypass the requirement for the *IM* gene product, likely because of compensating activities that make them less susceptible to photooxidation early in their development.

## PTOX Function

*IMMUTANS* was cloned in *Arabidopsis* by both map-based methods (Wu et al., 1999) and T-DNA tagging (Carol et al., 1999). The gene product (IM) was discovered to be a plastid membrane protein that is distantly related (37% amino acid similarity) to alternative oxidase (AOX), a mitochondrial inner membrane protein which functions in the alternative (cyanide-resistant) pathway of respiration, where it transfers electrons from ubiquinol to molecular oxygen (Wu et al., 1999). Central among its physiological functions, AOX is an important sensor of cellular redox balance (Giraud et al., 2008; McDonald, 2008). Similar to AOX, IM has quinol oxidase activity *in vivo* and *in vitro* and, consequently, it has been designated PTOX (Joët et al., 2002; Josse et al., 2003). PTOX is found in some cyanophages, which might serve as vectors for transfer of PTOX among cyanobacteria, but otherwise appears to be limited to oxygenic photosynthetic prokaryotes and eukaryotes, where it is found in all lineages (McDonald et al., 2011). *IM* is generally present as a single copy gene, although two copies are found in some cyanobacteria, red algae and green algae (Wang et al., 2009; Houille-Vernes et al., 2011). In chloroplasts, PTOX is bound to the stromal lamellae of thylakoids and is modeled as an interfacial membrane protein whose active site faces the stroma (Berthold et al., 2000; Joët et al., 2002; Lennon et al., 2003). It does not appear to be present in chloroplast envelope membranes.

Several functions have been ascribed to PTOX. The phytoene-accumulation phenotype of *im* led to the suggestion that PTOX serves as the terminal oxidase of an oxygen-dependent redox pathway that desaturates phytoene (Beyer et al., 1989; Mayer et al., 1990, 1992; Hugueney et al., 1992; Schulz et al., 1993; Nievelstein et al., 1995; Norris et al., 1995; Al-Babili et al., 1996). This pathway is thought to involve transfer of electrons from phytoene to plastoquinone (PQ) via PDS, forming ζ-carotene and plastoquinol (PQH_2_), and from PQH_2_ to molecular oxygen via PTOX, forming water and PQ (Carol et al., 1999; Wu et al., 1999; Rosso et al., 2009). Thylakoids of developing *im* plastids have over-reduced PQ pools (Rosso et al., 2009), and according to this pathway, the accumulation of phytoene in *im* can be explained by a decreased supply of PQ available to PDS, as suggested by Okegawa et al. (2010), and/or because electron transfer from phytoene into an over-reduced PQ pool is not energetically favorable (Rochaix, 2011). In addition to the phytoene-accumulation phenotype of mutants that lack PTOX (*im* in *Arabidopsis* and the orthologous *ghost* mutant in tomato) (Wetzel et al., 1994; Josse et al., 2000; Barr et al., 2004; Shahbazi et al., 2007), an involvement of PTOX in carotenogenesis is suggested by the close coordination between PTOX expression and carotenoid production in a number of systems, perhaps most strikingly in chromoplasts during the ripening of tomato, citrus, and pepper fruit (Josse et al., 2000; Barr et al., 2004). Another noteworthy example is the upregulation of PTOX expression in etiolated seedlings of *Arabidopsis* treated with paclobutrazol (PAC), an inhibitor of gibberellin biosynthesis; PAC causes an increase in carotenoid production (Rodríguez-Villalón et al., 2009).

It might be noted that all the enzymes of carotenogenesis are present in chloroplast envelopes, with the exception of PDS which is also found in thylakoids (Joyard et al., 2009). The involvement of PTOX in carotenogenesis is therefore puzzling given its apparent exclusive location in thylakoids (Lennon et al., 2003). This suggests that carotenoid intermediates are trafficked between the envelope and thylakoids, perhaps via vesicles, by connections between the envelope and thylakoids, or by carrier proteins (Adam et al., 2011; Ngaki et al., 2012). There might also be PTOX-independent mechanisms of plastoquinol oxidation in envelopes, such as PQH_2_ auto-oxidation (Khorobrykh and Ivanov, 2002), the activity of another plastoquinol oxidase (Buchel and Garab, 1995; Lajko et al., 1997; Joët et al., 2002), or perhaps a plastoquinol peroxidase (Casano et al., 2000; Rumeau et al., 2007).

*IM* mRNAs are ubiquitously expressed in *Arabidopsis*, and the anatomies of various plastid types are affected in *im* (Aluru et al., 2001), as well as in tomato *ghost* (Josse et al., 2000; Barr et al., 2004). However, a positive correlation between *IM* mRNA levels and carotenoid accumulation does not always hold, and *IM* is expressed highly in some tissues with low carotenoid accumulation (Aluru et al., 2001). Considered together, these observations argue that the role of PTOX in plastid metabolism is not limited to carotenogenesis. In fact, since its discovery over 13 years ago, it has become apparent that PTOX resides at the nexus of a growing number of redox pathways in the plastid. For instance, *in vitro* and *in vivo* studies have demonstrated that PTOX is the terminal oxidase of chlororespiration (non-photochemical reduction of the PQ pool) (Peltier and Cournac, 2002; Rumeau et al., 2007). Chlororespiration involves the transfer of electrons from NAD(P)H (and/or Ferredoxin) to PQ via a thylakoid NAD(P)H dehydrogenase (NDH) complex, and from PQH_2_ to molecular oxygen via PTOX (Peng et al., 2012). One purpose of chlororespiration is to help poise the redox state of the PQ pool during cyclic electron flow (CEF) around PSI (Trouillard et al., 2012). In addition to chlororespiration, PTOX mediates chromorespiration, the analogous process in chromoplasts (Josse et al., 2000; Barr et al., 2004; Rodrigo et al., 2004; Shahbazi et al., 2007).

Safety valves are photoprotective mechanisms that dissipate excess photons and electrons (Niyogi, 2000). They include non-photochemical quenching mechanisms and alternative electron acceptors that prevent over-reduction of photosynthetic electron carriers, thereby protecting PSI and PSII from photodamage (Aro et al., 1993; Allahverdiyeva et al., 2005). Soon after its discovery, Niyogi (2000) proposed that PTOX-mediated electron flow from PQH_2_ to O_2_ might act as a safety valve. Early support for this hypothesis came from studies showing that the green leaf sectors of *im* have morphological, biochemical, and molecular adaptations similar to plants acclimated to growth in high light, even when grown in permissive (low light) conditions (Aluru et al., 2001). Early studies also showed that *IM* mRNAs and proteins are significantly upregulated in high light conditions in antisense mutants of tobacco that lack both ascorbate peroxidase and catalase (Rizhsky et al., 2002). A number of experiments have since supported the safety valve hypothesis in systems as diverse as the alpine species *Ranunculus glacialis* and *Soldanella alpina* (Streb et al., 2005; Laureau et al., 2011); oat exposed to heat and high light (Quiles, 2006); mature green leaves and green fruit of the tomato *ghost* mutant subjected to high light stress (Shahbazi et al., 2007); wild species of *Brassica fruticulosa* versus the agricultural species *Brassica* *oleracea* adapted to both heat and high light (Díaz et al., 2007); the salt-stressed halophyte *Thellungiella* (Stepien and Johnson, 2009); and cold-acclimated Lodgepole pine (Savitch et al., 2010).

In addition to altered expression in response to environmental factors, PTOX is upregulated in various photosynthetic mutants, including the *gun4* mutant of *Chlamydomonas* and the tobacco *rbcL* and *psbA* deletion mutants. The *gun4* mutants lack GUN4, a regulatory subunit of Mg-chelatase, and enhanced PTOX activities in these mutants were suggested to play a physiological role in decreasing PSII excitation pressures (Formighieri et al., 2012). The *rbcL* mutants lack Rubisco and consequently two major electron sinks, CO_2_ fixation and photorespiration; it was proposed that elevated PTOX levels in these mutants reduce oxidative pressure on both PSI and PSII (Allahverdiyeva et al., 2005). In the *psbA* mutants, which lack the D1 protein of PSII, it was hypothesized that enhanced PTOX levels primarily support increased carotenoid production as a way to quench singlet oxygen generated by free LHCII accumulation in the mutant (Baena-González et al., 2003). Despite the growing number of examples of PTOX upregulation, *cis* and *trans* regulatory factors have yet to be reported.

The demonstration that PTOX serves as a safety valve in a particular system is not straightforward and must rely on more than gene expression data, since there are many ways of preventing over-reduction of the electron transport chain, such as upregulation of downstream electron sinks, as found in some cold-tolerant plants, or upregulation of the Mehler reaction (e.g., Gray et al., 1997; Savitch et al., 2000); alterations in PTOX expression might be a secondary effect. There are also some cases where PTOX does not act as a safety valve. For example, overexpression of *Arabidopsis* PTOX does not impart increased resistance to photoinhibition in mature tobacco leaves (Joët et al., 2002), nor does it regulate the redox state of the PQ pool during steady state photosynthesis in *Arabidopsis* (Rosso et al., 2006). These findings are consistent with the observation that the steady state flux of electrons through PTOX is relatively minor – estimated to be <2% of the flux through the photosynthetic electron transport chain (PETC) (Ort and Baker, 2002). On the other hand, these same studies showed that PTOX is important in preventing over-reduction of PSII acceptors during dark to light transients (Joët et al., 2002), and that it protects against PSI photoinhibition (Rosso et al., 2006). The latter has been confirmed in recent experiments showing that PTOX acts as a safety valve in *Arabidopsis* under stress conditions of low temperature and high light, where it protects against both PSII and PSI photoinhibition (Ivanov et al., 2012).

In this context it is important to note that in contrast to PSII, where there are a number of photochemical and non-photochemical mechanisms to avoid over-reduction of PSII acceptors, the repertoire is rather more limited for PSI and includes, most prominently, the water–water cycle (reviewed in Rumeau et al., 2007). PTOX might thus turn out to be an important mechanism to reduce electron pressure on PSI, as during stress when NADPH/ATP ratios are high and CEF around PSI is activated; in concert with NDH, PTOX might serve to poise the redox state of intersystem carriers by recycling electrons to PQ. This might also be the case during chloroplast biogenesis when it has been demonstrated that PTOX is a central regulator of thylakoid redox and PSII excitation pressure (Rosso et al., 2009).

Although not within the scope of this review, it might be mentioned that the functions described for PTOX in cyanobacteria and eukaryotic algae are similar to those in higher plants (reviewed by McDonald et al., 2011). These include carotenogenesis and response to diverse environmental stresses, such as high light, temperature, salt, iron, and phosphate deprivation (Moseley et al., 2006; Bailey et al., 2008; Wang et al., 2009). One difference is that cyanobacterial respiratory and PETCs share some components, which offers the possibility of PTOX control of both activities.

**Figure 1:**
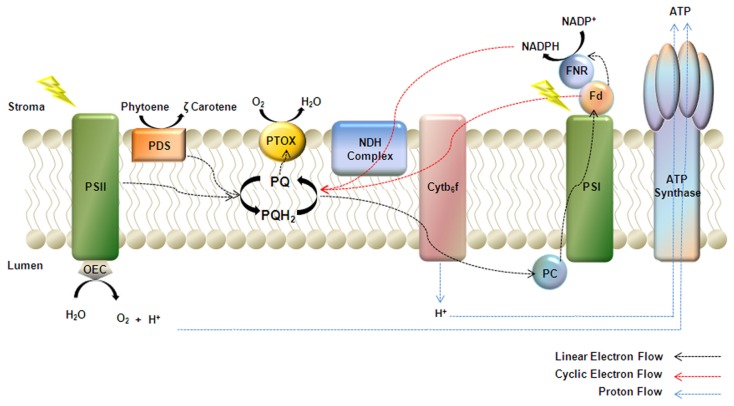
**Model of PTOX as a cofactor of phytoene desaturase (PDS)**. PTOX is a plastoquinol terminal oxidase that regulates the redox state of the plastoquinone pool (PQ) during early chloroplast biogenesis. Electrons from linear electron flow, cyclic electron flow mediated by either NDH or the Fd dependent PGR5 pathway, and the desaturation of phytoene, feed into the PQ pool. PTOX plays a pivotal role in transferring electrons from the PQ pool to molecular oxygen thus keeping the pool oxidized.

*In summary*, there is growing evidence that PTOX is a versatile terminal oxidase in chloroplast metabolism and that its primary physiological role varies from taxon to taxon. This might not be surprising, since PTOX is only one element of a large network of factors involved in stress tolerance, pigment biosynthesis, and photosynthetic control (regulation of ATP/NADPH ratios) (Kramer and Evans, 2011; Foyer et al., 2012). In such a network, it might be anticipated that the relative importance of a given element varies according to developmental and/or physiological context. For instance, PTOX might turn out to play a more significant regulatory role in PSI cyclic electron transport in bundle sheath cells of C4 plants than in mesophyll cells of C3 plants; in contrast to C3 plants, cyclic electron transport is a key regulator of ATP synthesis during C4 photosynthesis (Rumeau et al., 2007; Foyer et al., 2012). Given the ubiquity of PTOX expression, it might also be predicted that novel functions will be found for PTOX, especially in non-green plastids. A schematic diagram of PTOX in thylakoids is shown in Figure 1.

## Mechanism of Variegation: The Threshold Hypothesis

### Studies using mature leaves

Nearly all molecular and biochemical studies on *im* have been conducted using fully expanded rosette leaves; for comparative studies, green versus white sectors have been obtained by dissection or via fluorescence-activated cell sorting (FACS) (Wetzel et al., 1994; Meehan et al., 1996; Aluru et al., 2009). Alternatively, all-green *im* leaves have been produced by altering light conditions during early seedling development (Rosso et al., 2009).

**Figure 2:**
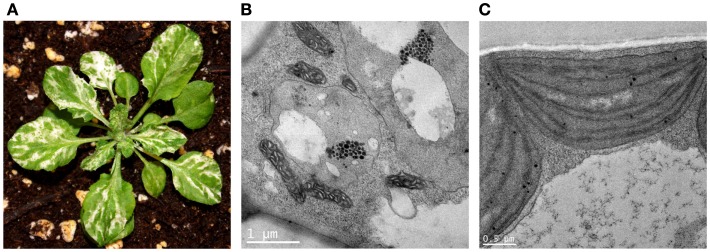
**Ultrastructural analysis of *immutans* (A)**. A representative *immutans* plant grown at 22°C under continuous illumination for 7 days at 5 μmol photons m^−2^s^−1^ followed by 3 weeks at 100 μmol photons m^−2^s^−1^. **(B)** Representative plastids from an *im* white sector. The plastids lack organized lamellar structures and contain vacuoles and numerous plastoglobules. **(C)** Representative chloroplast from an *im* green sector.

The notion that *im* variegation is due to a *threshold* came from two sets of observations. The first was that the white sectors of *im* are heteroplastidic, indicating that *im* behaves in a plastid autonomous manner, i.e., plastid phenotypes within a cell are determined independently in the *im* background. The second was that intermediate plastid phenotypes are not seen; plastids in the green sectors look similar to one another, as do abnormal plastids in the white sectors (Figure 2). Taken together with the finding that the white (but not green) sectors accumulate phytoene, the first iteration of the *threshold hypothesis* held that plastid phenotype in *im* is determined by the attainment of a threshold of PDS activity during chloroplast biogenesis: developing plastids with above-threshold activities are able to produce enough downstream, photoprotective carotenoids to form normal-appearing chloroplasts (green sectors), whereas plastids with below-threshold PDS activities are deficient in colored carotenoid production and susceptible to photooxidation (white plastids and sectors) (Wetzel et al., 1994; Wetzel and Rodermel, 1998). This hypothesis was later refined after *IM* was cloned and its function was defined (described in greater detail later).

Chloroplast biogenesis involves the differentiation of a small number of proplastids in the shoot apical meristem into chloroplasts early in leaf primordial development (Mullet, 1988). This process is accompanied by several rounds of plastid division (Robertson et al., 1995; Pyke and López-Juez, 1999). In C3 plants like *Arabidopsis*, leaf expansion along the proximal-distal axis results from cell divisions at the base of the leaf, while cell divisions at the center of the leaf and extending outward cause lateral expansion (Pyke et al., 1991; Van Lijsebettens and Clarke, 1998). Hence, the oldest chloroplasts are present in cells at the tips and margins of the leaf, whereas the youngest ones are found in cells at the base of the leaf and at the midveins. Although plastid number depends on cell size (Possingham and Lawrence, 1983), a typical *Arabidopsis* meristem cell contains a dozen or so proplastids whereas a mesophyll cell contains from 100 to 120 mature chloroplasts (Mullet, 1988; Pyke and López-Juez, 1999). The numbers of plastids in *im* green and white sectors have not yet been reported.

Wetzel et al. (1994) performed light-shift experiments to determine when PTOX activity is first required following germination. These studies showed that cotyledon pigmentation (all-green, all-white, or variegated) is influenced by the intensity of light (low or high) perceived between 0 and 24 h after seed coat breakage; the intensity perceived before or after this interval did not matter. During this period, proplastids differentiate into chloroplasts within the developing cotyledon (Mansfield and Briarty, 1996). This indicates that PTOX plays a crucial role in early chloroplast biogenesis.

**Figure 3:**
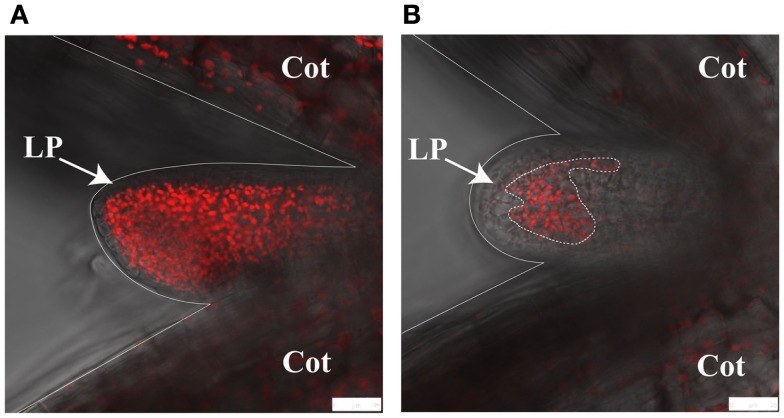
**Confocal microscopy of leaf primordia**. The images show first leaf primordia (LP) and flanking cotyledons (Cot) from wild type **(A)** and *immutans* **(B)**. Images were captured by taking a confocal image of autofluorescence (red) and overlaying it with a non-confocal transmitted light image (gray). Plants were grown at 22°C under continuous illumination for 3 days at 5 μmol photons m^−2^s^−1^ followed by 2 days at a light intensity 100 μmol photons m^−2^s^−1^. White scale bars represent 25 μm.

We have recently exploited confocal microscopy to visualize *im* variegation during leaf primordia development. Figure 3 shows the red autofluorescence from chloroplasts in cells of wild type and *im* leaf primordia; each primordium is located between the two cotyledons. The figure dramatically illustrates that *im* cotyledons and primordia have fewer chloroplasts than wild type, and that *im* variegation develops very early in leaf development.

### Studies using greening as a model system

Etioplasts in dark-grown angiosperm seedlings have prolamellar bodies (PLB) that contain abundant ternary complexes of protochlorophyllide (PChlide), PChlide reductase (POR), and NADPH (Solymosi and Schoefs, 2010). Upon exposure to light, PChlide is converted to chlorophyllide (Chlide) and the membranes of the PLB disperse to form thylakoids and organized grana (Adam et al., 2011). Most of the protein components of the photosynthetic apparatus are present in etioplasts, and hence de-etiolation (greening) primarily involves the coordinated synthesis and assembly of pigments and pigment-binding proteins to form functional electron transport chains (von Zychlinski et al., 2005; Blomqvist et al., 2008; Kanervo et al., 2008). Photosynthetic competence is attained in *Arabidopsis* ∼5 h after light induction at 22°C and 100 μmol photons m^−2^s^−1^ (Rosso et al., 2009).

Using *Arabidopsis* de-etiolation as a model system, Hüner’s group found that white sector formation in *im* is positively correlated with elevated excitation pressures during early greening, irrespective of whether excitation pressures are modulated by light and/or temperature (Rosso et al., 2009). Excitation pressure is a relative measure of the reduction state of *Q*_A_, the first stable electron acceptor of PSII (Dietz et al., 1985; Hüner et al., 1998), and is measured by chlorophyll *a* fluorometry (Krause and Weis, 1991). The elevated excitation pressures in *im* were accompanied by delayed biogenesis of the thylakoid membrane, as monitored by the abundance of protein markers of chloroplast development (Rosso et al., 2009). Given these considerations, our current working model of *im* variegation assumes that above-threshold excitation pressures predispose developing plastids to photooxidation, whereas *im* plastids with below-threshold excitation pressures have the capacity to develop into normal chloroplasts. This model is based on the twin notions of plastid autonomy and plastid heterogeneity. We surmise that excitation pressures vary from plastid-to-plastid in the developing leaf primordium because of intrinsic differences in plastid biochemistry caused, for example, by gradients in the leaf, sometimes steep, of light and of determinants of light capture, and use (Smith et al., 1997), including cell-specific circadian rhythms that dictate reactive oxygen species (ROS) scavenging capacities (Velez-Ramirez et al., 2011). Such heterogeneity appears to be widespread and has recently been reported in species as diverse as trees (Solymosi et al., 2012). One advantage of this model is that it can account for the phytoene-accumulation phenotype of *im*, as well as for the ultrastructural defects of *im* white plastids. For instance, one of the primary targets of over-reduction might be membrane biogenesis since a prominent effect of ROS formation is lipid peroxidation of polyunsaturated fatty acids (Møller et al., 2007). A lack of plastid galactolipids coupled with a lack of stabilizing carotenoids might impede membrane formation, giving rise to the large vesicles seen in *im* white plastids (Figure 2).

*In summary*, our current model of *im* variegation proposes that the pattern of green/white sectoring is a reflection of the pattern of leaf development in C3 plants, coupled with a heterogeneity in plastid-to-plastid excitation pressure during early cell and plastid development, i.e., the chaotic sectoring in the mature leaf is established in the leaf primordium. Recent support for the threshold hypothesis has come from the *altered APX2 expression 13* (*alx13*) variegation mutant of *Arabidopsis* (Woo et al., 2011).

## Compensating Factors

Because the green sectors of *im* contain morphologically normal chloroplasts, we have proposed that they arise from plastids that escape the effects of the *im/im* mutation early in chloroplast biogenesis, presumably by the action of compensating factors that affect excitation pressure thresholds, directly or indirectly. Two approaches have been used to gain insight into these factors: (1) molecular characterization of green versus white *im* sectors; and (2) identification and characterization of second-site suppressors of *im* variegation. These approaches will be summarized briefly.

### How do the green and white sectors of *im* differ?

The green leaf sectors of *im* have higher photosynthetic rates than wild type leaves, monitored either by rates of O_2_ evolution or ^14^CO_2_ uptake (Aluru et al., 2001, 2007). They also have elevated chl *a/b* ratios and enhanced chlorophyll amounts; FACS analyses have revealed these increases are due to elevated chloroplast numbers (per unit volume) rather than to enhanced amounts of chlorophyll per chloroplast (Meehan et al., 1996). Accompanying these changes, the anatomies of the green leaf sectors are reminiscent of leaves adapted to growth in high light conditions, e.g., they are thicker than normal due to enlarged air spaces, mesophyll cells, and epidermal cells (Aluru et al., 2001). On the other hand, the white leaf sectors have a normal thickness, but their palisade cells fail to expand. This perturbation is consistent with an impairment in retrograde signaling that regulates leaf developmental programming (Rodermel, 2001; López-Juez, 2009; Ruckle and Larkin, 2009). We have made a similar proposal to explain reduced fruit size and disruptions in pericarp tissue morphogenesis in the tomato *ghost* mutant (Barr et al., 2004).

Consistent with elevated rates of photosynthesis, the green leaf sectors have increased Rubisco and sucrose phosphate synthase (SPS) activities, enhanced starch and sucrose pool sizes, and an altered pattern of carbohydrate partitioning that favors sucrose over starch (Aluru et al., 2007). By contrast, the white sectors accumulate low levels of sucrose and have increased acid invertase activities. These observations suggest that sucrose moves along a gradient from the green to the white cells, where it is hydrolyzed and used for growth. It is therefore possible that photosynthetic rates in the green sectors are controlled, in part, by sink demand, and that the elevation of photosynthetic rates in the green sectors is part of a growth strategy to compensate for reductions in total source tissue (Aluru et al., 2007).

Global transcriptomics experiments have revealed that some of the differences between the green and white *im* sectors are likely due to alterations in transcript abundance (Aluru et al., 2009). In particular, genes for photosynthesis and photosynthesis-related processes are repressed in *im* white tissues, while genes for sucrose catabolism, transport, mitochondrial electron transport, and fermentation are induced. This suggests that energy is derived via aerobic and anaerobic metabolism of imported sugar in the *im* white cells, in accord with findings from the biochemical studies, discussed above. The profiling studies also showed that oxidative stress response genes are generally induced in both the green and white *im* tissues, but that some stress genes are significantly more upregulated in the green than white sectors. These genes are targets for investigation as potential compensating factors.

### Second-site suppressors of *im* variegation

The above studies have been conducted with mature leaves, and although they have provided valuable information about *im*, they are limited in their usefulness regarding information about the primary lesion in *im* because they describe developmental, physiological, and biochemical states that represent long term adaptations to the lack of PTOX in the leaf. They do not provide information about the primary alterations that occur when the mutation first becomes active during chloroplast biogenesis and when compensating mechanisms might first come into play.

Given that all alleles of *im* isolated to date are null (Carol et al., 1999; Wu et al., 1999), an attractive strategy to identify compensating factors is to take advantage of *Arabidopsis* genetics to characterize second-site suppressors of *im* variegation, i.e., second-site mutations that reverse the *im* defect, generating all-green plants. These factors would be anticipated to define elements and/or processes that are able to substitute for or bypass the requirement for PTOX activity during chloroplast biogenesis. One advantage of using a genetic approach to identify these factors is that the early stages of chloroplast development are difficult to access by other approaches (biochemistry, molecular biology). Presumably, compensating factors that are active during early chloroplast biogenesis would permit the desaturation reactions of carotenogenesis to occur in a PTOX-independent manner under high light conditions, e.g., by other oxidases or by PTOX-independent pathways of PQH_2_ re-oxidation coupled to PDS (discussed earlier). Shahbazi et al. (2007) provided early support for this idea on the basis of experiments showing that phytoene did not accumulate following exposure of mature green leaf or fruit tissues of the *ghost* mutant to high light: carotenoid synthesis occurred normally in the absence of PTOX. Later in chloroplast development we assume that compensating factors include elements of the PETC downstream of the PQ pool that are capable of oxidizing plastoquinol, thus obviating the need for PTOX.

In this context, it might be noted that early studies showing an O_2_ requirement for PDS activity were based on *in vitro* systems from non-photosynthetic chromoplasts (Beyer et al., 1989; Mayer et al., 1990). Interestingly, an “oxidoreductase fraction” was proposed to act as an intermediate between PDS and O_2_ in these systems (Beyer et al., 1989). We suggest that this O_2_ requirement reflects a need for PTOX activity in the absence of compensating factors, such as photosynthetic electron transport. This would be consistent with the general conclusion that PTOX is required for carotenogenesis during chloroplast biogenesis and stress, but not during steady state photosynthesis (Rosso et al., 2006, 2009).

#### AOX2 and AOX1a suppressors

To identify *im* suppressors, we have conducted EMS and activation-tagging mutagenesis of *im* and screened for mutant plants with a non-variegated phenotype. Molecular characterization of one suppressor line (designated *ATG791*) has been reported (Fu et al., 2012). This line is all-green and, surprisingly, carries an activation-tagged (overexpressed) version of mitochondrial AOX2. *Arabidopsis* contains five members of the AOX gene family, all of which are thought to be exclusively mitochondrial in location (AOX1a-d and AOX2); AOX2 is a low abundance, seed-specific member of this family (Saisho et al., 1997, 2001; Clifton et al., 2006; Winter et al., 2007; Polidoros et al., 2009).

We found that AOX2 is targeted to chloroplast thylakoids of *ATG791*, where its activity replaces that of PTOX in the desaturation steps of carotenogenesis, i.e., it rescues the phytoene-accumulation defect of *im*, restoring carotenoid biosynthesis to normal. This restoration is accompanied by a normalization of excitation pressures, suggesting that the two processes are linked (Fu et al., 2012). It is therefore likely that elevated carotenoids in the suppressor lines are directly responsible for rescue of the variegation phenotype, and hence for recovery of the capacity to undergo normal chloroplast biogenesis. Results similar to *ATG791* were obtained when AOX2 was overexpressed using the CaMV 35S promoter, or when *AOX1a* – the most highly and ubiquitously expressed member of the *Arabidopsis* AOX gene family, was re-engineered to target the plastid; overexpressed AOX1a is found exclusively in mitochondria (Clifton et al., 2006; Winter et al., 2007; Polidoros et al., 2009). In both cases, the *im-*induced defects in phytoene-accumulation and chloroplast biogenesis were reversed.

Further experiments with the AOX2 and AOX1a overexpression lines showed that chloroplast-localized AOX2, but not AOX1a, formed monomers and dimers in the thylakoid membrane, reminiscent of AOX regulation in mitochondrial inner membranes. In addition, both proteins accumulated as higher molecular weight complexes in thylakoids, though these complexes differed in size and number between the two lines. Interestingly, photosynthetic activities, as monitored by chlorophyll fluorescence, were not generally perturbed in the overexpression lines, nor was growth altered. This suggests that the presence of AOX1a and AOX2 complexes in thylakoids does not significantly perturb steady state photosynthesis, at least under non-stress conditions. Our operating assumption is that the suppressor lines define novel electron transport paths in the chloroplast.

AOX2 has previously been localized to mitochondria (Saisho et al., 2001), and because it was imported into chloroplasts using its own transit peptide in the *AOX2* overexpression lines, as well as in transient expression assays, our current working hypothesis is that AOX2 is a dual-targeted protein that functions in plastids to supplement PTOX activity during the early events of chloroplast biogenesis. Although the idea that AOX2 is normally an *im*-compensating activity needs further confirmation, the ability of AOX1a to substitute for PTOX in the correct physiological and developmental contexts is a dramatic example of the capacity of a mitochondrial protein to replace the function of a chloroplast protein, and illustrates the plasticity of the photosynthetic apparatus. This is all the more remarkable given the striking differences in regulation, substrate-specificity (ubiquinol in mitochondria versus plastoquinol in plastids) and phylogenetic distance between AOX and PTOX (McDonald et al., 2011; Fu et al., 2012). It will therefore be of great interest to explore further how chloroplast AOX1a and AOX2 mediate redox reactions during photosynthesis.

#### *pgr5* and *crr2–2* suppressors

Shikanai’s group has long been studying mechanisms of PSI cyclic electron transport, a process that contributes to ΔpH formation across the thylakoid, but not to NAD(P)H accumulation, thus providing a way of manipulating ATP/NAD(P)H ratios (Shikanai, 2007). Okegawa et al. (2010) reported that two mutants perturbed in this process – *crr2-2* (*chlororespiratory reduction*) (Hashimoto et al., 2003) and *pgr5* (*proton gradient regulation 5*) (Munekage et al., 2002) are able to suppress *im* variegation. The *pgr5* mutant is defective in electron transport, has a reduced ΔpH, an over-reduced stroma (a decreased ATP/NADPH), an oxidized P700^+^, and a loss of qE (Munekage et al., 2002; DalCorso et al., 2008; Okegawa et al., 2008). The *crr2-2* mutant, on the other hand, is defective in NDH activity due to aberrant expression of the plastid *ndhB* gene (Hashimoto et al., 2003); it has photosynthetic characteristics similar to *pgr5* (Okegawa et al., 2010). Neither of these mutants has a chloroplast development phenotype (Munekage et al., 2004).

Okegawa et al. (2010) assumed that *crr2-2* and *pgr5* define separate pathways of PSI cyclic electron transport, and given this assumption, the goal of their experiments was to examine genetic interactions between these pathways and PTOX, all of which share the PQ pool. The observation that loss of either one of these pathways (in *crr2-2* or *pgr5*) or both (in *crr2-2/pgr5*) is able to suppress *im* variegation can be explained by reduced excitation pressures during early chloroplast development, due to a decreased flux of electrons into the PQ pool. The converse suppression by *im* of the photosynthetic defects in *crr2-2* and *pgr5* is more problematic to explain, but might be due to altered ATP/NADPH ratios and activation of the water–water cycle in an *im* background (Okegawa et al., 2010).

One factor complicating an understanding of the mechanism of *im* suppression in these studies is that considerable controversy surrounds the function of PGR5 (reviewed in Kramer and Evans, 2011). The only agreement seems to be that PGR5 plays a role in fluctuating light conditions (Tikkanen et al., 2010). Although further studies will be needed to define the interactions between PTOX, PGR5, and NDH, it is clear from the present data that interactions occur between these three components very early in chloroplast development. All three are also present in etioplasts (Aluru et al., 2001; Long et al., 2008; Peng et al., 2008), perhaps to help poise the PQ pool for photosynthesis.

*In summary*, suppressor analysis holds great promise as a tool to understand the function of PTOX and mechanisms of compensation that occur in its absence. The data in this paper high light the significance of *im* suppressor analysis for understanding pathways and interactions that mediate early chloroplast development, and they include the identification of novel proteins (AOX2) and pathways (PSI cyclic electron transport).

## Conflict of Interest Statement

The authors declare that the research was conducted in the absence of any commercial or financial relationships that could be construed as a potential conflict of interest.
